# The Oral Virome in Dental Caries: From Ecological Disruptors to Therapeutic Opportunities

**DOI:** 10.4014/jmb.2604.04028

**Published:** 2026-08-24

**Authors:** El-Sayed Khafagy, Gillan I. El-Kimary, Ahmed Al Saqr, Wael A. H. Hegazy

**Affiliations:** 1 Department of Pharmaceutics, College of Pharmacy, Prince Sattam bin Abdulaziz University, Al-Kharj 11942, Saudi Arabia; 2 Department of Preventive Dental Sciences, College of Dentistry, King Faisal University, Al-Ahsa, Saudi Arabia; 3 Department of Microbiology and Immunology, Faculty of Pharmacy, Zagazig University, Zagazig 44519, Egypt; 4 Pharmacy Program, Oman College of Health Sciences, Muscat 113, Oman

**Keywords:** Oral virome, Bacteriophages, Dental caries, Phage therapy, Enzybiotics, Ecological dysbiosis

## Abstract

Dental caries results from ecological dysbiosis of oral biofilms driven by frequent sugar exposure, yet the role of viruses, particularly bacteriophages, in this process remains underexplored. This review synthesizes current evidence on how the oral virome contributes to caries pathogenesis and examines emerging phage-based therapeutic strategies. The oral cavity harbors diverse viral communities, including eukaryotic viruses (herpesviruses, papillomaviruses) and prokaryotic viruses (bacteriophages). While herpesviruses like EBV, CMV, and HSV-1 do not directly demineralize teeth, they may act as ecological stressors by modulating host immunity, altering gingival crevicular fluid composition, and promoting inflammation that favors cariogenic bacterial activity. Bacteriophages function as key ecological regulators through “kill-the-winner” dynamics, maintaining microbial diversity by preying on dominant species. However, temperate phages can horizontally transfer genes encoding acidogenicity, biofilm formation, or antibiotic resistance, potentially accelerating dysbiosis. Metagenomic studies reveal distinct salivary phage signatures associated with caries, suggesting diagnostic potential. Preclinical evidence demonstrates that lytic phages targeting *Streptococcus mutans* (*e.g.*, SMHBZ8, ΦAPCM01) disrupt biofilms *in vitro* and prevent caries in *ex vivo* and murine models. Phage-derived enzymes (endolysins, depolymerases) and peptides offer additional precision antimicrobials with minimal commensal disruption. Despite promising advances, significant translational gaps remain, including limited well-characterized phages, insufficient polymicrobial model testing, and absence of human clinical trials. Integrating virome ecology into caries management represents a paradigm shift from pathogencentric approaches toward ecosystem-based therapeutics, with phages offering targeted, self-replicating, and evolutionarily dynamic tools for restoring oral health.

## Introduction

Dental caries (tooth decay) is a chronic, multifactorial disease where acids produced by bacteria in dental plaque dissolve tooth minerals, usually in response to frequent sugar intake and disturbed oral biofilm balance [1]. Cavities are the most prevalent non-communicable disease globally, affecting billions of individuals across all age groups. Their significance extends far beyond a mere dental issue, impacting overall health, quality of life, and socioeconomic well-being [1, 2]. Meta-analysis studies involving large populations indicate a strong correlation between caries and periodontitis, often occurring in the same individuals and even on the same teeth [3, 4]. Individuals with periodontitis exhibit a heightened propensity for developing tooth decay, encompassing both crown and root caries [3, 5]. Gingival inflammation and bleeding are more prevalent in areas affected by untreated caries [4, 6]. Untreated caries progresses through the enamel and dentine layers to reach the pulp chamber, leading to pulpitis and pulp necrosis. Subsequently, bacteria can spread through the root canal to the root apex, causing apical periodontitis and abscess formation [7, 8]. Next-generation sequencing reveals the shared presence of several bacterial species between deep caries dentine and infected root canals. This suggests that caries bacteria may contribute to the development of primary apical periodontitis [9]. The most serious concern is the potential for root canal infections to create portals for bacteria to enter the bloodstream. These infections have been implicated as contributors to infective endocarditis and other systemic inflammation, particularly in high-risk heart patients [7, 8].

The oral microbiome contains bacteria, fungi, archaea, protozoa, and viruses. “Oral virome” most likely refers to oral viruses. The virome comprises the community of viruses (primarily bacteriophages that infect bacteria, along with some human viruses) residing in the oral cavity and engaging in interactions [10, 11]. Regrettably, viruses have received less attention in research compared to bacteria within the oral microbiome [12, 13]. In this review, we aimed to elucidate the role of the oral virome in the development of dental caries and explore the potential of phages as therapeutic agents for caries treatment.

## The Caries Ecosystem Revisited

The comprehension of dental plaque’s role in caries has undergone a significant conceptual transformation, culminating in the contemporary ecological perspective that positions the disease as a microbial dysbiosis (Fig. 1). For much of the 20th century, the Specific Plaque Hypothesis dominated, positing that caries was an infectious disease caused by a finite number of specific pathogens, most notably *Streptococcus mutans* [14, 15]. This view logically led to therapeutic strategies aimed at the targeted elimination of these “cariogenic” organisms. However, the persistence of disease and the recognition of the immense diversity of the oral microbiome exposed limitations in this model. It failed to adequately explain why these putative pathogens could be found in low numbers at healthy sites or why disease severity did not always correlate directly with their counts [1, 16]. This paradigm shift was formalized in 1994 with Philip D. Marsh's seminal proposal of the Ecological Plaque Hypothesis (EPH) [14, 17]. This framework fundamentally redefined caries not as a simple infection but as a consequence of a destabilized microbial ecosystem. The EPH proposes that in oral health, the plaque biofilm exists in a state of dynamic equilibrium, or homeostasis, where a diverse community of microorganisms is maintained in balance by host defenses (*e.g.*, salivary flow, buffering capacity) and limited nutrient availability [14, 15, 17]. Caries occurs when sustained environmental pressures, such as the frequent intake of fermentable carbohydrates, disrupt this balance. The resultant increased acid production lowers the plaque pH, creating a selective environment that favors acidogenic and aciduric bacteria, such as *S. mutans*, *Lactobacilli*, and *Bifidobacteria*, while inhibiting organisms associated with health. Therefore, the disease-associated community is a product of ecological selection, not merely the overgrowth of a single exogenous pathogen [14, 16]. The EPH has been further refined to elucidate the caries process as a metabolic transformation that broadens the scope of caries etiology beyond bacterial involvement. It establishes a conceptual framework wherein the entire ecosystem including its viral components must be taken into account [14].

Building upon this ecological framework, a critical and previously under-researched question arises: how do viruses, particularly bacteriophages, impact the intricate equilibrium between homeostasis and dysbiosis within the dental plaque ecosystem [18]. This central inquiry reframes the caries process by introducing viruses as active ecological forces, not mere bystanders. The conventional bacteriological perspective on the EPH necessitates the integration of the virome, a fundamental component of the oral microbiome [18, 19]. These viruses, primarily bacteriophages that infect bacteria, possess the unique potential to act as powerful top-down ecological regulators. A crucial question arises; do specific phage profiles contribute to maintaining a stable, health-associated plaque by inhibiting the proliferation of potentially cariogenic species. Conversely, do environmental pressures like sugar intake select for or activate prophages that confer ecological advantages to aciduric bacteria, thereby accelerating the drive towards dysbiosis. By interrogating how viruses contribute to both the resilience of healthy plaques and the pathogenesis of diseased ones [20]. In the subsequent sections, we will endeavor to elucidate a novel dimension of caries etiology, wherein the equilibrium between health and disease is orchestrated through a multifaceted interplay of oral bacteria and their viral adversaries.

## Viruses in the Oral Cavity

The oral cavity hosts a highly diverse oral virome comprising viruses that infect human cells and abundant bacteriophages that infect bacteria [21-23]. Several viruses can reside or replicate in the oral cavity without causing symptoms, while others can lead to distinctive lesions or contribute to chronic inflammatory and neoplastic disorders [13, 24, 25]. Metagenomic analyses of nearly 2,800 oral samples have cataloged over 48,000 non-redundant viral genomes, significantly expanding the known diversity and revealing numerous predicted phages and eukaryotic viruses [21]. Viral communities differ between saliva, tongue, teeth, and gingiva, showing strong niche specialization within an individual [21, 22].

### The Indirect Influencers: Eukaryotic Viruses

Reviews highlight frequent detection of herpesviruses, human papillomaviruses (HPV), enteroviruses, paramyxoviruses (measles, mumps), and Human immunodeficiency virus (HIV), among others [13, 24-26]. These viral infections may induce multiple effects on the oral microbiome. Viral infections establish latent or persistent infections in oral epithelium, salivary glands, or lymphoid tissue [27, 28]. Consequently, these infections cause primary or recurrent ulcers, vesicles, erythema, or hyperkeratotic lesions [24, 26]. Furthermore, reactivation occurs in immunosuppressed states (*e.g.*, HIV infection), where oral lesions frequently mirror disease progression [24, 25, 28]. Oncoviruses, including HPV, Epstein-Barr virus (EBV), and Kaposi’s sarcoma-herpesvirus (KSHV), are increasingly implicated in the development of oral and oropharyngeal cancers. Their oncogenic potential is attributed to their persistent infection, immune evasion, disruption of cell cycle and apoptosis pathways, dysbiosis, and coinfections [23, 29].

Notably, human herpesviruses, such as EBV, human cytomegalovirus (CMV), and herpes simplex virus-1 (HSV-1), are among the most studied viruses in the oral cavity [30, 31]. Their presence is frequently detected in oral tissues and inflammatory lesions, indicating the establishment of lifelong latent infections that can reactivate [30-33]. Studies indicate a significantly higher proportion of diseased oral sites, such as severe periodontitis lesions, harboring these viruses compared to healthy sites, suggesting a potential pathogenic role in oral inflammatory diseases. Although their direct involvement in caries is less well-established than in periodontitis, evidence suggests they can influence host immune responses and gingival crevicular fluid (GCF), potentially impacting cariogenic conditions [30, 33, 34].

Herpesviruses, EBV and CMV, infect immune cells such as B lymphocytes, monocytes, and macrophages, as well as gingival epithelial cells. This infection leads to immunosuppression or dysregulation, which are essential for controlling bacterial biofilms [30, 31, 35]. This can impair the host’s ability to control bacterial populations, allowing overgrowth of cariogenic bacteria, including those involved in caries and periodontitis [30, 31, 35, 36]. Herpesviruses and periodontopathic bacteria often co-infect the same sites, with evidence of synergistic interactions that exacerbate tissue destruction and inflammation [18, 30, 31, 34-36]. This synergistic effect may indirectly create conditions, such as heightened inflammation and compromised immune surveillance, that promote the persistence and activity of cariogenic bacteria.

Furthermore, reactivation of herpesviruses in the periodontium can trigger the release of pro-inflammatory cytokines (*e.g.*, IL-1β, TNF-α, prostaglandins), which promote inflammation and tissue breakdown [30, 35, 37]. EBV infection of gingival fibroblasts specifically increases inflammatory cytokine production, further aggravating local immune responses [37]. GCF and periodontal pockets serve as reservoirs for herpesviruses, with higher viral loads correlating with disease severity [30, 31]. The presence of these viruses in GCF alters its composition, leading to an increase in inflammatory mediators and potentially affecting the local environment in ways that promote cariogenic bacterial activity [30, 31, 35]. Additionally, the induced inflammation enhances the flow of GCF, thereby providing a more abundant source of proteins and glycoproteins. These nutrients can subsequently serve as nourishment for acidogenic bacteria present in the adjacent dental plaque [38]. This inflammation can lead to an increase in the volume of GCF and its concentration of proteolytic enzymes, such as Matrix Metalloproteinase-9 (MMP-9). MMP-9 is a host-derived enzyme that breaks down collagen in dentin. This altered GCF can then come into contact with the plaque biofilm at the gingival margin, potentially influencing its ecological dynamics and delivering enzymes that synergize with bacterial acids to degrade tooth structure [39, 40]. In summary, although herpesviruses do not directly cause tooth decay, they are hypothesized to function as indirect ecological stressors. Individuals with higher herpesvirus loads or compromised immune responses may be more susceptible to shifts in the oral microbiome, which could lead to increased cariogenic potential [18, 30, 31, 35] (Fig. 2).

### The Main Actors: Bacteriophages

The oral cavity contains one of the body’s most diverse microbiomes, with ~700–770 bacterial species and 50–100 billion bacterial cells in an average adult mouth. These bacteria exhibit a hierarchical structure, with a relatively small group of phyla and genera dominate most healthy mouths and occupy specific niches such as teeth, tongue, gingiva and saliva. Across healthy individuals, six phyla make up ~96% of oral bacteria: *Firmicutes*, *Actinobacteria*, *Proteobacteria*, *Bacteroidetes*, *Fusobacteria*, and *Spirochaetes* [41, 42]. This “core” structure exhibits remarkable conservation despite inter-individual variations [41, 42]. *S. mutans* (phylum *Firmicutes*) is widely regarded as the primary cariogenic pathogen in dental plaque [43, 44]. However, *S. mutans* is generally low in relative abundance throughout the oral cavity and is often undetectable, even in individuals with caries [45, 46]. Sequencing studies show that healthy saliva and plaque are dominated by multiple genera (*e.g.*, *Streptococcus* spp., *Neisseria*, *Veillonella*, *Prevotella*, and *Actinomyces*), not specifically by *S. mutans* [47-49]. So, *S. mutans* is not the numerically dominant bacterium in the oral cavity, but it is one of the most important when conditions favor caries.

Bacteriophages, crucial regulators in dental plaque, are potential precision tools against cariogenic pathogens. However, the oral phageome remains largely unexplored, with limited phage discoveries against key pathogens like *S. mutans* [50]. Bacteriophages dominate healthy and diseased plaque and saliva; in periodontitis, subgingival viromes are shifted toward lytic myoviruses, suggesting viruses may help drive ecological change in dysbiotic biofilms [22]. Phage isolation from oral samples presents significant challenges, with only a limited number of successful isolations reported. Furthermore, the majority of phages exhibit a restricted host range [51]. To date, only a limited number of phages that specifically target *S. mutans* have been isolated and comprehensively characterized (Table 1). In the subsequent sections, we intend to elucidate how phages influence the ecology of the plaque ecosystem.

**Lytic Phages vs. Lysogenic Phages.** Metagenomic studies indicate the coexistence of lytic and lysogenic phages in saliva and plaque, thereby continuously shaping bacterial diversity and biofilm structure [56-58]. The phage influence is multifaceted; through predatory lytic cycles, they can directly modulate bacterial population densities; through lysogeny, they can horizontally transfer genetic traits. Lytic phages, which infect bacteria, replicate swiftly, and induce host cell lysis releasing progeny phages. These phages function as top-down predators, potentially constraining the excessive growth of *S. mutans* [51, 52, 54]. Temperate phages possess the ability to transition between the lytic and lysogenic cycles. During the lysogenic cycle, the phage genome integrates into the bacterial chromosome, functioning as a prophage. This integration allows for replication alongside the host and potentially introduces novel traits, such as enhanced immunity against subsequent infections or heightened pathogenicity such as increased acid production, enhancing biofilm formation and antibiotic resistance [45, 55, 59, 60]. Temperate phages serve as genetic reservoirs and can either diminish or amplify cariogenicity through lysogenic conversion and stress-induced transitions to lytic cycles [19, 45, 56] (Fig. 3).

Particularly, temperate phages facilitate horizontal gene transfer (HGT) among oral bacteria, spreading genes that may increase cariogenic potential. This genetic exchange can occur between different species within the oral cavity, accelerating the evolution of more pathogenic or resilient strains [56, 59]. For instance, lysogenic phages are prevalent among *Enterococcus faecalis* and other *Streptococcus* species and *Actinomyces* species. Prophage-containing strains often show altered phenotypes, such as enhanced biofilm formation and increased persistence, which can contribute to caries risk [56, 61]. Shifts in the abundance and activity of these phages are linked to changes in the oral microbiome that favor cariogenic bacteria [56].

**Phages as Ecological Engineers of the Plaque Ecosystem.** Beyond the direct lysis of individual cells, the most profound impact of bacteriophages on dental caries lies in their capacity to act as top-down regulators of the entire plaque microbial community. Through continuous predation and co-evolutionary dynamics, phages selectively pressure bacterial populations, thereby sculpting the plaque's composition, diversity, and functional output. This ecological role positions phages not merely as bacterial killers but as fundamental architects of the oral microenvironment, capable of tipping the balance between health and disease [57, 62] (Fig. 4).

A central ecological model governing phage-bacteria interactions is the “kill-the-winner” dynamic. This principle posits that phages preferentially target the most abundant, actively replicating bacterial taxa within a community [57, 62]. In the context of dental plaque, a pioneer or early colonizer that establishes dominance, such as *S. mutans* under a high-sugar diet, becomes a primary target for specific lytic phages. Their predation prevents any single species from monopolizing the niche, thereby preserving microbial diversity [11, 57]. A diverse plaque community is a hallmark of health, as it fosters metabolic stability and resilience against pathogen overgrowth. The presence of cross-infecting phages, which show negative correlations with key pathogens and positive correlations with commensals, exemplifies this stabilizing role. By suppressing “winner” populations, phages indirectly create ecological space for less competitive but potentially beneficial bacteria to thrive, preserving the homeostasis envisioned in the EPH [11, 62-64]. Consequently, the selective pressure exerted by phages fosters an ongoing evolutionary arms race. Bacteria develop a range of defense mechanisms, encompassing receptor modification, CRISPR-Cas systems, and abortive infection mechanisms [65, 66]. The emergence of phage-resistant bacterial clones has direct implications for plaque ecology. The rise of a phage-resistant subpopulation can lead to a fundamental shift in the dominant strains within a species. This change alters the genetic and functional landscape of the plaque biofilm, potentially impacting its collective acidogenicity or biofilm integrity [45, 55, 59, 60]. This dysbiotic shift towards caries may be facilitated by a breakdown in predatory phage control. If phages fail to regulate emerging cariogenic “winner” populations, or if temperate phages disseminate acidogenicity genes, they can accelerate disease progression [63, 64]. Nevertheless, harnessing this ecological principle presents a sophisticated strategy for caries management. By selectively targeting keystone cariogenic pathogens, we can open a promising avenue to overcome these pathogens, as we will demonstrate in the subsequent sections.

## Therapeutic and Diagnostic Applications of Phages in Controlling Cariogenic Bacteria

### Phage-based Viral Signatures in Saliva as Caries Biomarkers

Recent studies indicate that salivary phage communities change systematically with caries status, suggesting diagnostic potential and indicating that could a promising diagnostic tool. The alterations in the oral phageome of disease-associated phages infecting key oral bacteria, such as *Streptococcus*, *Neisseria*, and *Porphyromonas*, are observed across various conditions, including caries, periodontitis, and systemic diseases. This study explicitly highlights the potential of phage signatures as indicators of oral and general health [52, 58, 67]. Long-read metagenomics in saliva reveals a diverse, partly novel phage community, including *Streptococcus* phage groups and jumbo phages carrying antimicrobial resistance and other accessory genes, underscoring the informational richness of the salivary virome for biomarker development [68]. Tang and Baker conducted a metagenomic analysis of children with severe caries compared to healthy, which controls revealed a significant disparity in beta diversity of salivary viromes (predominantly phages) between the two groups, irrespective of other metadata. Specific viral operational taxonomic units (vOTUs) predicted to infect *Neisseria*, *Haemophilus*, and *Streptococcus* were enriched in health, whereas phages predicted to infect *Veillonella*, *Saccharibacteria*, *Prevotella*, and *Scardovia* were enriched in caries. These disease-linked vOTUs tended to co-occur with bacterial taxa associated with the same health or disease status, supporting a biologically coherent shift in the phage–bacteria network rather than random noise [10].

A broader meta-analysis integrating 199 salivary and plaque metagenomes from six caries cohorts similarly found that virome composition differs between caries and health in both children and adults, with hundreds of differentially abundant vOTUs in supragingival plaque and adult saliva. Phages were mainly *Siphoviridae*, *Myoviridae*, *Podoviridae*, and *Autographiviridae*, and carried auxiliary genes for carbohydrate metabolism, antibiotic resistance, and immune modulation that could enhance cariogenic potential of their hosts [69]. Another study of severe early childhood caries reported decreased alpha diversity and increased beta diversity of the salivary/oral phageome, with family-level shifts (*e.g.*, increased *Podoviridae* and decreased *Streptococcus* phages) and functional changes in carbohydrate-metabolism genes compared to controls [67]. Current salivary caries biomarker work focuses almost entirely on host and bacterial molecules (proteins, enzymes, cytokines, pH, flow rate), not phages [70-72].

Unfortunately, current findings are predominantly cross-sectional discovery studies. The heterogeneity in sampling, analytical methods, and population factors has impeded the validation of robust, chairside tests. Consequently, multi-marker panels are likely necessary [72, 73]. The aforementioned virome studies revealed statistically significant case-control differences at both community and vOTU levels. Notably, there was a reproducible association between specific phage taxa and health or caries across various datasets, particularly for certain groups. Furthermore, the co-occurrence of phage markers with known cariogenic or health-associated bacteria was observed. However, none of these studies report clinically ready predictive models (*e.g.*, sensitivity and specificity) utilizing phage abundances alone. Additionally, there is a lack of longitudinal validation demonstrating that baseline phage patterns can predict future caries incidence.

### Phage Therapy for Cariogenic Biofilms

The translation of phage biology into a viable therapeutic strategy for dental caries rests on the promise of developing targeted, precise, and ecologically sound treatments. The rationale is compelling: unlike broad-spectrum antimicrobials, phages offer species-specific activity that could selectively reduce *S. mutans* (Table 1), the primary cariogenic pathogen, while preserving the protective commensal oral flora. Furthermore, their ability to replicate at the site of infection and penetrate the protective biofilm matrix addresses two critical weaknesses of conventional therapies [19, 74]. Phage therapy is increasingly explored for oral biofilm-associated diseases, including dental caries, though evidence is still preclinical and limited compared with other infection sites. Cariogenic biofilms are notoriously resistant to conventional antimicrobial treatments due to their complex structure and protective extracellular matrix [19]. While phage therapy has shown efficacy against various biofilm-forming pathogens *in vitro* and in animal models, direct evidence specifically targeting cariogenic biofilms remains limited [74]. Most available research focuses on foundational studies of phage–biofilm interactions in oral environments or extrapolates from related models such as periodontitis or device-associated biofilms [19]. This review synthesizes the current state of *in vitro*, *ex vivo*, and animal model evidence for phage therapy against cariogenic biofilms.

***In Vitro* Studies: Demonstrating Specificity and Biofilm Disruption.** Multiple *in vitro* studies demonstrate that phages isolated from saliva or dental waste can effectively inhibit *S. mutans* biofilm formation and reduce the viability of established biofilms. These phages often show high host specificity and rapid lytic activity, making them suitable candidates for targeted therapy [51, 54, 55, 75, 76]. The isolation and characterization of phage φAPCM01 provided foundational evidence, showing it could rapidly lyse planktonic *S. mutans* and, crucially, dismantle mature biofilms, achieving a greater than 5-log reduction in viable cells. This demonstrated that phages are not merely effective against free-floating bacteria but can actively penetrate and destroy the structured, protective biofilm communities central to caries pathogenesis [51, 54]. The isolation of φKSM96 is particularly noteworthy as a temperate phage from *S. mutans*. While temperate phages can integrate into the host genome, φKSM96 exhibited strong lytic activity, significantly inhibiting biofilm formation and altering the ecological balance in favor of non-cariogenic species in mixed cultures. This highlights that temperate phages are not merely genomic passengers but can be potent antimicrobial agents and tools for future genetic engineering [55].

Zhu *et al*. demonstrated that the use of lytic phages isolated from the oral cavity generally resulted in substantial reductions in biofilm biomass and viability against key cariogenic and periodontal species. These findings support the notion that host-adapted oral phages with appropriate lytic properties can effectively target oral biofilms *in vitro* [75]. *In vitro* models provide a platform for evaluating phage efficacy individually or in conjunction with other therapeutic agents, such as probiotics and antibiotics. Phage therapy has demonstrated a consistent ability to significantly reduce biofilm biomass and metabolic activity, often surpassing other modalities in terms of specificity and efficacy [51, 53, 54, 76]. Additionally, *in vitro* studies provide mechanistic insights into the phenomenon of phage-resistant bacterial survivors. These studies reveal that such survivors may exhibit reduced fitness and virulence, and in certain instances, become more susceptible to antibiotics. This suggests a potential synergy between phage and antibiotic therapies [77, 78]. It is noteworthy that *in vitro* evidence suggests that appropriately selected lytic phages, potentially in combination, can significantly disrupt oral and cariogenic biofilms. However, clinically realistic multispecies models of plaque are still uncommon [75, 77-79]. Examples of studies that have demonstrated the efficacy of phages against cariogenic biofilms are summarized in Table 2.

**Animal and *Ex Vivo* Model Trials.** Animal studies are the critical bridge between laboratory findings and potential human clinical trials, testing phage therapy in a complex, living system. Evidence specific to dental caries is still confined to small *in vitro* studies and a handful of oral disease phage reports; no robust rodent caries models with phage treatment have yet been systematized in the literature base provided [75]. *Ex vivo* and *in vivo* studies on animals demonstrated that phages possess the ability to control biofilm-associated infections, encompassing those affecting mucosal and hard tissue surfaces [74, 79]. These findings provide potential analogues for caries. *In vivo* studies investigating the efficacy of phages in eliminating cariogenic pathogens and their associated biofilms are limited (Table 2). While most animal studies focus on *S. mutans*, phage therapy has also been tested against other oral pathogens and in models of periodontitis and periapical disease, showing broad-spectrum potential [55, 75].

For instance, the comprehensive caries-specific evidence originates from a study by Wolfoviz-Zilberman *et al*., which employed the lytic phage SMHBZ8. In this study, hemi-mandibles from euthanized mice subjected to caries-promoting conditions were utilized *ex vivo*. The findings revealed that treatment with SMHBZ8 phage (in suspension or a sustained-release varnish) effectively prevented the formation of carious lesions, a result corroborated by chlorhexidine treatment. This observation was further validated in a live mouse model of dental caries. Mice administered with SMHBZ8 phage suspension exhibited no signs of demineralization upon clinical and micro-CT examination, whereas untreated, infected controls developed substantial lesions. Notably, this study also pioneered a practical delivery method by formulating the phage into a hydroxypropyl-cellulose varnish for sustained release. This formulation demonstrated equal efficacy and non-toxicity to host cells [53].

*In vivo* studies have demonstrated several advantages, including the confirmation of the safety of phage therapy, as evidenced by the absence of observed toxicity in treated animals. Delivery systems, such as phage suspensions or gels, are being investigated to enhance retention and efficacy in the oral cavity [53, 75]. However, there is a significant translational gap; oral/cariogenic indications are underrepresented, standardized cariogenic biofilm models (multispecies plaque, saliva, enamel/dentin) are lacking, and controlled animal caries studies are scarce [52, 75, 79]. Despite these promising results, the path to clinical application requires addressing several key challenges identified in the literature. The limited availability of well-characterized phages against cariogenic bacteria presents a significant challenge. To address this, it is imperative to expand the isolation and characterization of phages from diverse oral and environmental sources, thereby ensuring comprehensive coverage [75]. Most studies focus on single-species biofilms; future research should address the complexity of polymicrobial dental plaque [77]. Furthermore, methodological inconsistencies in phage-biofilm experimentation impede comparison and reproducibility. For instance, inadequate reporting of phage titers and the use of overly simplistic *in vitro* conditions that poorly mimic the oral environment [53, 75, 78]. The future of phage therapy may lie in the genetic modification of phages like φKSM96 to enhance their lytic activity, broaden host range, or equip them with additional biofilm-degrading enzymes [54, 75]. In addition to enhancing oral delivery (*e.g.*, varnishes, rinses, gels), conducting long-term safety and toxicology studies, and designing robust clinical trials to establish efficacy in humans.

**Phage Enzymes: Enzybiotics.** Phage lysins, also referred to as “enzybiotics”, are bacteriophage-derived enzymes that degrade bacterial cell walls. Phage lysins (peptidoglycan hydrolases) function by cleaving peptidoglycan in bacterial cell walls, resulting in rapid cell lysis and death. This mechanism is particularly effective against Gram-positive bacteria, such as the primary agent in dental caries, *S. mutans* [79, 94, 95]. Fortunately, lysins demonstrate exceptional specificity towards their bacterial targets, thereby minimizing the disruption to the beneficial oral microbiome when compared to traditional broad-spectrum antibiotics [95]. Furthermore, the likelihood of bacteria developing resistance to lysins is low, as these enzymes specifically target essential and highly conserved cell wall structures [79, 95].

Enzybiotics can penetrate and disrupt biofilms and are effective against metabolically inactive persister cells, which are often resistant to conventional antibiotics [79, 95]. In addition to peptidoglycan hydrolases, polysaccharide depolymerases degrade the extracellular polymeric substances (EPS) that constitute the structural framework of dental biofilms. By breaking down EPS, depolymerases weaken the matrix, rendering bacteria more susceptible to removal and antimicrobial agents [52, 77]. Depolymerases constitute a diverse class of enzymes that encompass Glycoside Hydrolases. These enzymes play a crucial role in breaking down glucan and fructan polymers, such as dextran and mutan, which serve as essential structural components of the EPS produced by streptococci [96-98]. Additionally, Dispersin B is a depolymerase that specifically targets poly-N-acetylglucosamine (PNAG), a matrix polysaccharide found in certain biofilms [99]. Alginate Lyases, another class of depolymerases, targ*et al*ginates present in *Pseudomonas* biofilms. While their relevance to cystic fibrosis is more pronounced compared to caries, these enzymes illustrate the general mechanism of depolymerization [100]. Depolymerases, particularly glycoside hydrolases, directly dismantle the EPS scaffold [101]. Once the EPS matrix is compromised by depolymerases, the underlying bacterial cells become exposed. This allows endolysins to access and bind to their peptidoglycan target on the cell wall [100]. This novel and highly specific approach presents a promising solution for combating dental caries bacterial infections (Table 2).

Preclinical and early applied research substantiates the potential of enzybiotics in oral care. Engineered and purified lysins have shown potent activity against *S. mutans* biofilms in both laboratory and animal models, significantly reducing biofilm mass and viability [79, 102]. A notable example is LysP53, a recombinant lysin formulated as a mouthwash. An *in vitro* study demonstrated that LysP53 at a concentration of 4 μM exhibited potent bactericidal activity against primary cariogenic pathogens like *S. mutans*, *S. sobrinus*, and *Acinetobacter naeslundii*, while showing poor activity against common oral commensal streptococci. When applied to established biofilms, the LysP53 mouthwash effectively reduced viable bacterial counts and disintegrated the biofilm structure [90]. Enzybiotics can be combined with phages or antibiotics for enhanced antibiofilm effects and to prevent the emergence of resistance [79, 103]. The combination of phage therapy and matrix-degrading enzymes can enhance biofilm removal, as enzymatic breakdown of the matrix allows phages and antimicrobials to penetrate deeper into the biofilm [52, 97, 98]. For instance, enzymes such as mutanase, β-glucanase, and DNase have demonstrated the ability to destabilize dental biofilms by targeting key matrix components, facilitating biofilm removal and potentially reducing caries risk [98, 104, 105]. The clinical translation of enzybiotics is advancing, with regulatory authorities granting approvals for specific indications, such as MRSA bacteremia, endocarditis and skin infections [95]. This could serve as an inspiration for research towards the development of enzybiotic preparations for the treatment of dental infections. Advancements in protein engineering hold the potential to revolutionize the production of chimeric and modified lysins, enhancing their solubility, stability, and activity against a broader spectrum of oral pathogens [94, 106]. Enhancing the delivery of dental preparations, particularly topical applications via varnish, gel, or mouth rinse, is the most logical and direct method to achieve a high localized concentration and minimize systemic distribution [75, 98]. A comparison between phage therapy, enzybiotics and conventional antimicrobials is summarized in Fig. 5.

**Phage-Derived Peptides.** Phage-derived peptides are synthetic or recombinant oligopeptides, typically ranging in length from 10 to 50 amino acids, whose sequence and function are identified or engineered through phage display technology [107]. Endolysin-derived peptides (EDPs) are cationic peptides designed from the cell wall-binding domains (CBDs) or selected catalytic regions of phage endolysins. Their primary function is to target and perturb the bacterial cell wall. These peptides can integrate into and disrupt the cytoplasmic membrane, leading to the leakage of cellular contents and cell death. Alternatively, they can retain enzymatic activity that interferes with peptidoglycan assembly, potentially triggering the activation of autolysins and resulting in bacterial death [108, 109]. Additionally, structural protein-derived peptides are mimic sequences derived from phage tail fibers, baseplate proteins, or other structural components involved in host recognition and attachment. These peptides can interfere with bacterial adhesion or biofilm formation [109].

Phage display enables the identification of peptides with high affinity for specific oral pathogens. For example, peptides like GA-C16G2 exhibit rapid and selective killing of *S. mutans*, with minimal effects on commensal species and low cytotoxicity to human cells [110-112]. Peptides that bind *S. mutans*, *S. oralis*, and *Lactobacillus casei* have demonstrated the ability to inhibit biofilm formation by these bacteria, a key factor in caries development [110, 113]. These peptides can prevent the establishment and maturation of cariogenic biofilms, often by binding to bacterial surfaces and interfering with adhesion or biofilm matrix formation [111-113]. Some phage-derived peptides are engineered to be activated by environmental cues, such as acidic pH found in cariogenic biofilms, ensuring selective action against pathogenic bacteria while sparing commensals [111, 114]. Dual-sensitive peptide nanoparticles (*e.g.*, pHly-1 NPs) have shown superior efficacy to traditional agents like chlorhexidine in animal models, reducing caries without harming oral microbiota diversity or tissues [111].

The utilization of phage-derived peptides aligns with the ecological plaque hypothesis by providing several advantages. Their specificity minimizes disruption to the health-associated oral microbiome and also reduces the window for adaptive resistance [115, 116]. Phage-derived peptides showed good stability in saliva and low toxicity, supporting their potential for clinical use [110, 111]. As small, synthetic molecules, they can be easily modified to enhance stability in the oral environment (resistance to proteolysis), improve penetration, or increase potency. They are also amenable to large-scale, cost-effective synthesis. Peptides can be incorporated into dental varnishes, gels, mouth rinses, or even tooth-binding delivery systems for sustained, localized release [113]. Despite their potential, translating phage-derived peptides to clinical practice necessitates robust *in vivo* caries models. Ultimately, human clinical trials are essential to validate their safety and therapeutic efficacy.

## Future Directions

Despite the promising advances in understanding the oral virome and the therapeutic potential of bacteriophages, several important challenges must be addressed before these approaches can be translated into routine clinical practice. First, the isolation, genomic characterization, and functional evaluation of additional phages targeting key cariogenic pathogens are required to expand the currently limited therapeutic repertoire. Second, future studies should employ standardized polymicrobial biofilm models that more accurately reproduce the complex oral ecosystem, incorporating saliva as well as enamel and dentin substrates to better predict clinical performance.

Furthermore, the development of optimized topical delivery systems, including varnishes, gels, mouth rinses, and sustained-release formulations, will be essential for maximizing phage stability, retention, and efficacy within the oral cavity. Comprehensive preclinical investigations evaluating pharmacodynamics, long-term safety, immunogenicity, and potential ecological effects on the commensal oral microbiome are also needed. Finally, well-designed randomized clinical trials are required to establish the efficacy, safety, and clinical applicability of phage therapy, phage-derived enzymes, and phage-derived peptides for the prevention and treatment of dental caries.

Advances in synthetic biology and phage engineering may further enhance therapeutic efficacy by expanding host range, improving biofilm penetration, and enabling the delivery of biofilm-degrading enzymes or other anti-virulence molecules. In parallel, continued development of virome-based biomarkers may facilitate early diagnosis, individualized risk assessment, and precision therapeutic strategies, ultimately integrating the oral virome into personalized caries management. Table 3 categorizes the current evidence into well-supported concepts, emerging evidence, and areas that remain speculative or require further investigation.

## Conclusion

The conceptual evolution of dental caries from a specific infectious disease to an ecological dysbiosis has fundamentally expanded our understanding of its etiology, yet the viral component of the oral microbiome has remained largely in the shadows. This review has synthesized emerging evidence positioning the oral virome not as a passive bystander but as an active ecological force capable of tipping the balance between oral health and disease. Eukaryotic viruses, particularly herpesviruses, may function as indirect stressors by modulating host immunity and creating inflammatory microenvironments that favor cariogenic bacterial activity. Bacteriophages, through lytic predation and lysogenic conversion, serve as top-down regulators of plaque community structure, potentially preserving diversity via “kill-the-winner” dynamics or accelerating dysbiosis by disseminating fitness-enhancing genes. The identification of distinct salivary phage signatures associated with caries status opens diagnostic avenues, while preclinical studies demonstrating phage efficacy against *S. mutans* biofilms provide proof-of-concept for therapeutic applications. However, the translational pathway from laboratory discovery to clinical practice remains fraught with challenges. The limited repertoire of well-characterized phages targeting oral pathogens, methodological heterogeneity across studies, scarcity of physiologically relevant polymicrobial biofilm models, and absence of human clinical trials collectively temper premature optimism. Phage-derived enzymes and peptides offer complementary advantages, including stability, scalability, and precision that may accelerate clinical adoption while circumventing some regulatory hurdles associated with live phage therapeutics. Looking forward, realizing the therapeutic potential of the oral virome will require: (1) expanded isolation and genomic characterization of phages targeting keystone cariogenic species; (2) development of standardized multispecies biofilm models incorporating enamel/dentin substrates; (3) rigorous long-term safety and toxicology studies; (4) innovative delivery systems (varnishes, gels, rinses) optimized for the oral environment; and (5) well-designed clinical trials establishing efficacy in human populations. As we increasingly recognize the oral cavity as an integrated ecosystem rather than a battleground of individual pathogens, phages and their derived products offer evolutionarily informed tools for restoring microbial balance, a therapeutic philosophy aligned with the ecological realities of caries pathogenesis.

## Figures and Tables

**Fig. 1:**
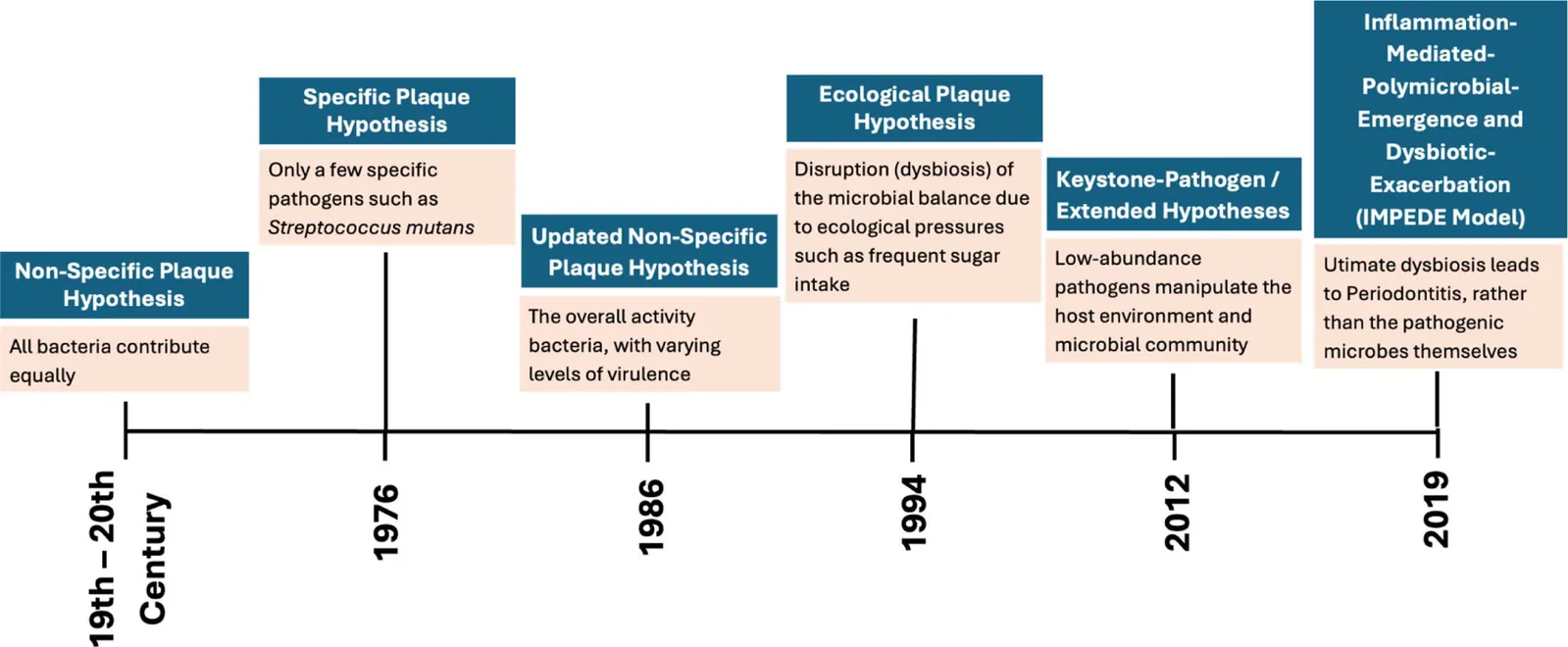
Hypotheses elucidate the progression of plaque and dental caries.

**Fig. 2:**
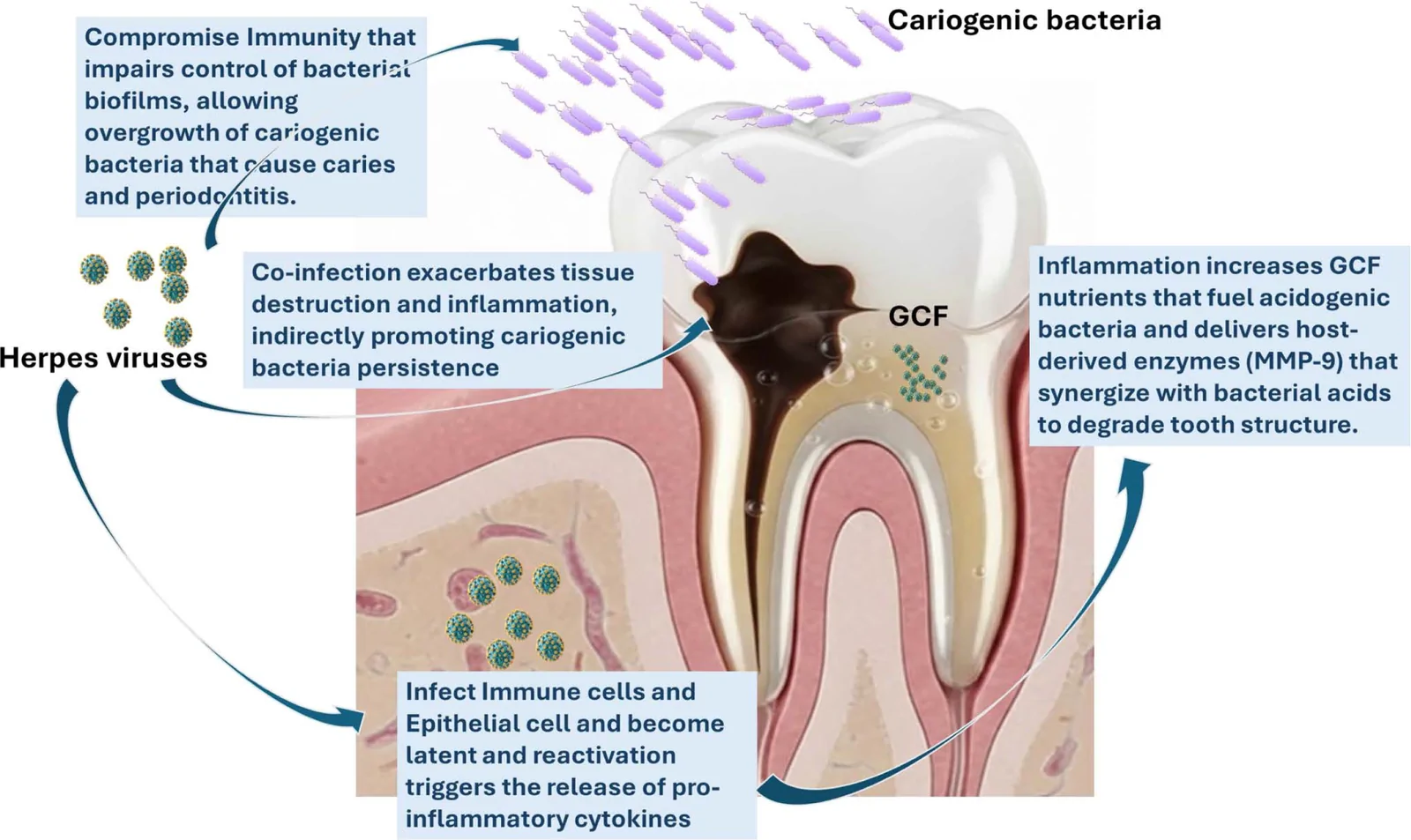
The indirect ecological stress of Herpes viruses. Herpes viruses such as HSV-1, CMV and EBV could increase the cariogenic potential. GCF: gingival crevicular fluid.

**Fig. 3:**
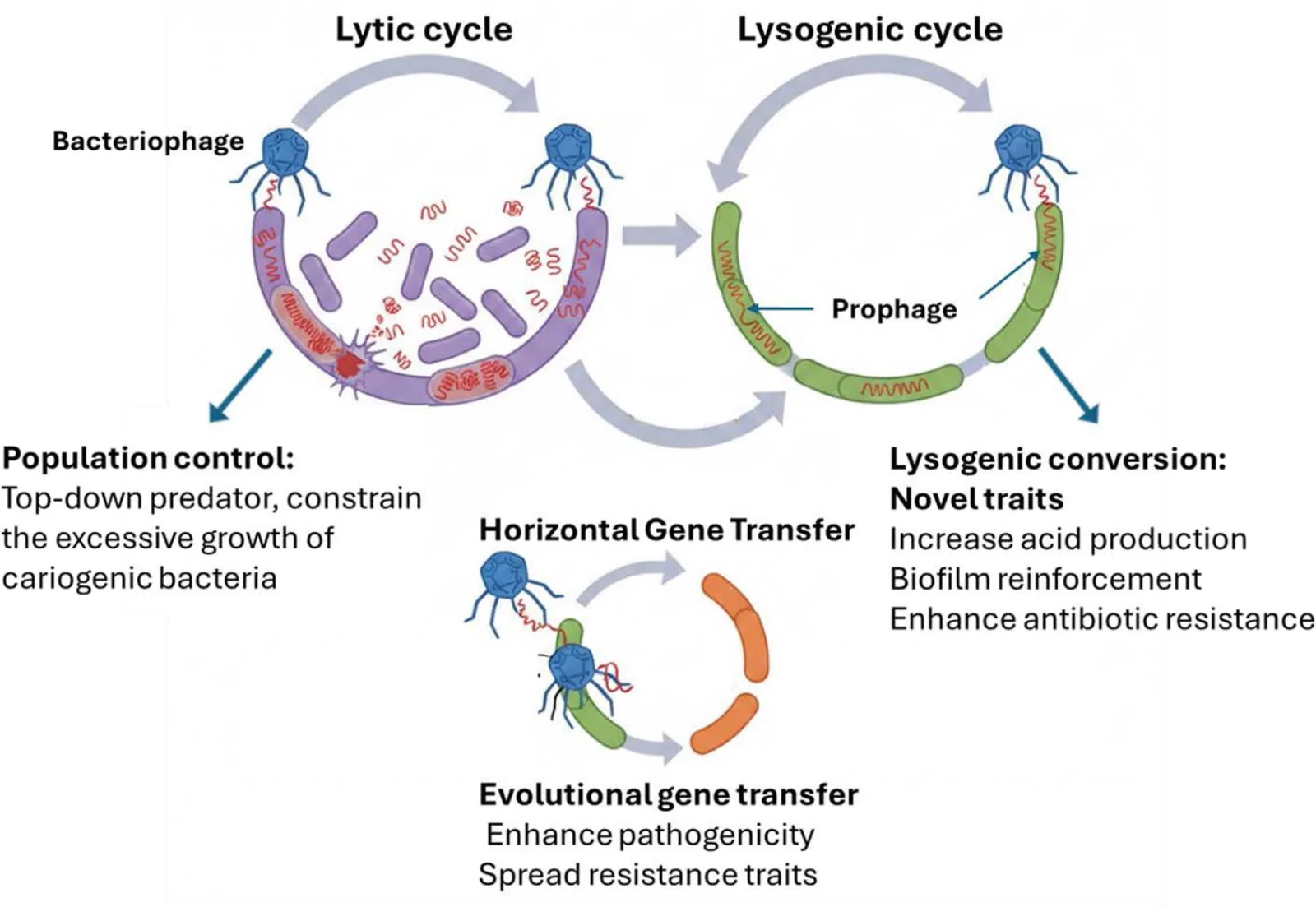
Phage dynamics in oral bacteriome. In the lytic cycle, virulent phages reduce bacterial growth, whereas in the lysogenic cycle, temperate phages can confer cariogenic traits.

**Fig. 4:**
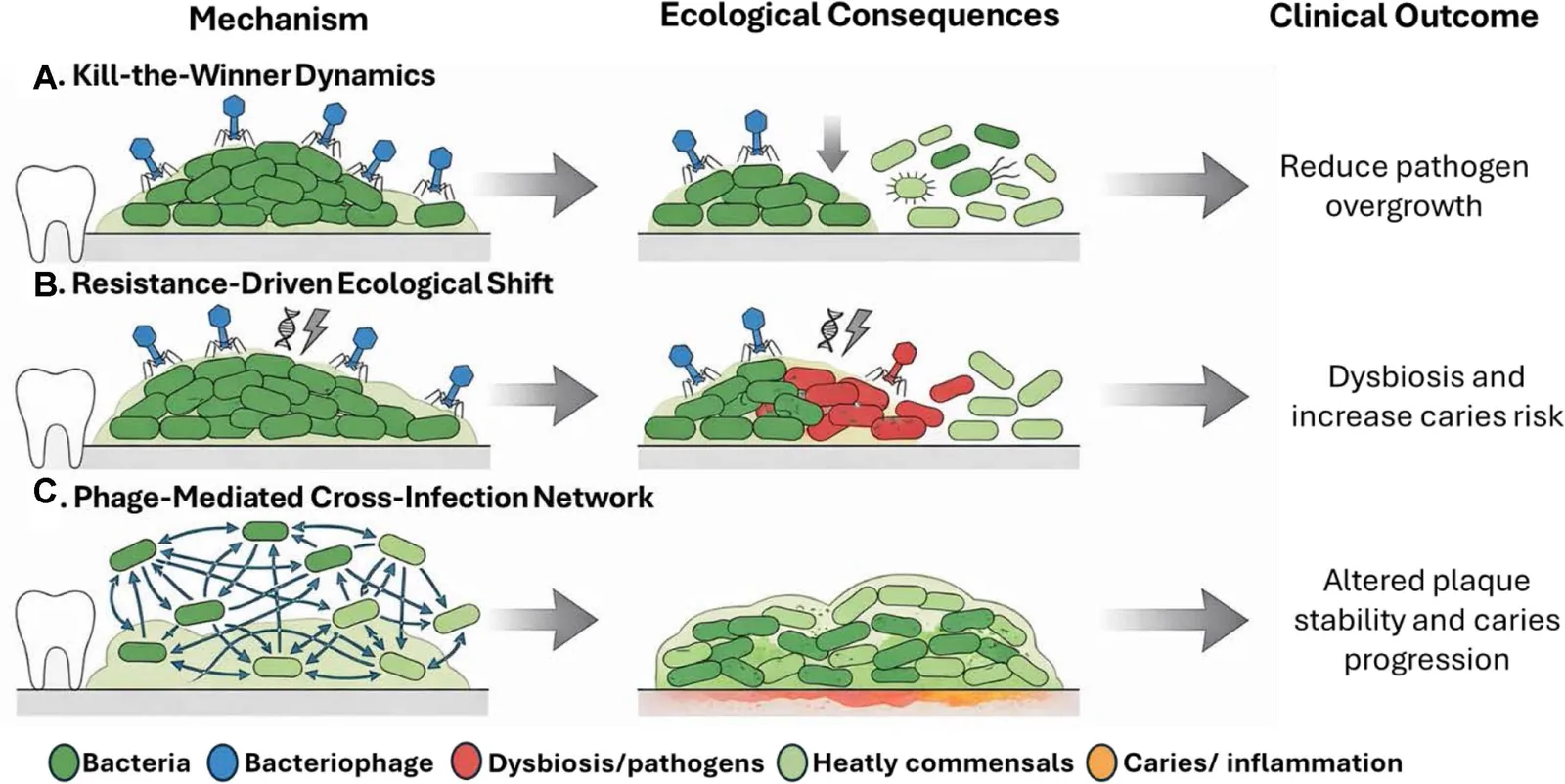
Phage-driven mechanisms shape oral microbial community structure and caries risk.

**Fig. 5:**
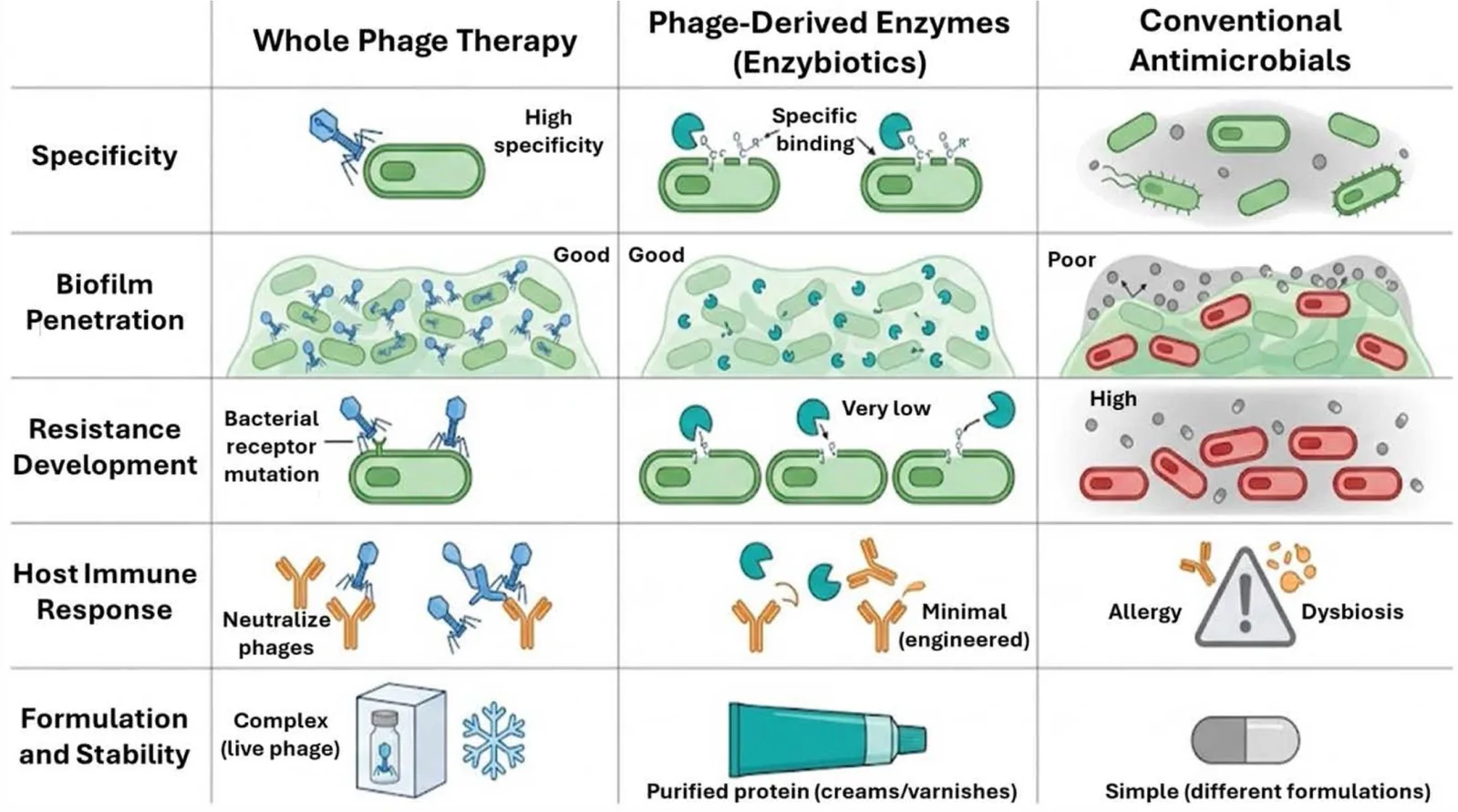
Comparison between therapeutic profiles; whole phage, phage-derived enzymes and traditional antimicrobials.

**Table 1:** Key *S. mutans* phages.

Phage	Life cycle	Key effects on cariogenic *S. mutans*	References
M102/M102AD	Lytic	Targets serotype C, strong lysis, defined lysis cassette, reduces biofilm	[51-53]
ΦAPCM01	Lytic	Strong killing in broth and artificial saliva, reduces biofilm	[51, 52]
SMHBZ8	Lytic	Kills planktonic and biofilm prevents caries in dentin and mouse models, a key step toward clinical relevance.	[53, 54]
φKSM96	Temperate	Broad serotype killing; inhibits growth and biofilms; a template for genetic engineering.	[52, 55]

**Table 2:** *In vitro*, *in vivo*, and *ex vivo* phage therapy for cariogenic studies.

Model type	Phage or phage-derived agent	Target bacteria / site	Main outcome on caries / cariogenic biofilm	Citations
*In vitro* human cariogenic dentin model	SMHBZ8 lytic phage (Siphoviridae)	*S. mutans* (cariogenic dentin from human teeth)	Marked reduction of *S. mutans* load in cariogenic dentin; phage more effective than antibiotic in reducing bacterial load in dentin model; activity reduced at pH <4 or >9; decreased medium acidification when added to sucrose-supplemented cultures	[54]
*In vitro* jaw (*ex vivo*-like) murine model	SMHBZ8 phage (suspension; varnish sustained-release)	*S. mutans* on dissected mouse hemi-mandibles under caries-promoting conditions	Phage in suspension or sustained-release formulation prevented demineralization and carious lesion formation; jaws resembled healthy and chlorhexidine-treated controls by clinical and μCT assessment	[53]
*In vivo* mouse caries model	SMHBZ8 phage suspension (oral swab)	*S. mutans* oral infection in mice	Significant inhibition of demineralization and carious lesion development; prevention of caries comparable to conventional measures; no reported toxicity in macrophage cell tests	[53]
*In vitro* *S. mutans* biofilm	ΦAPCM01	*S. mutans* biofilm	≥5-log CFU/mL reduction in viable cells in biofilm; effect persisted ≥48 h	[55]
*In vitro* screening (isolation only)	Unnamed *S. mutans* phage from dental plaque	*S. mutans* isolates from plaque	Single phage with narrow host range isolated; demonstrated feasibility of obtaining *S. mutans*-specific phages from dental plaque; no caries or animal model yet	[80]
*In vitro* caries-associated pathogens (non-mutans)	vB_SauM-EG-AE3 and vB_EfaP-EF01 lytic phages	*Staphylococcus aureus* and *Enterococcus faecalis* from dental caries lesions	Strong lytic activity and complete inhibition of growth in broth at MOI 10–10^3^ over 1–24 h; suggests potential to control caries-associated opportunists, but no enamel/caries outcome measured	[81]
*Ex vivo* human dentin (endodontic) & *in vitro*	vB_EfaS_ZC1	Vancomycin-resistant *Enterococcus faecalis* root-canal biofilm	Strong lytic and antibiofilm activity *in vitro*; in *ex vivo* infected human dentin, phage (alone or with propolis) significantly reduced mature *E. faecalis* biofilm vs. conventional NaOCl irrigation	[82]
*Ex vivo* infected root canals & *in vitro* plates	Unnamed *E. faecalis* phage	*E. faecalis* biofilm in 96-well plates; root canal dentin	*In vitro*: OD reduced from 0.95±0.16 (control) to 0.17±0.04 (phage), comparable to 1% NaOCl; *ex vivo*: CFU ~135,000 in control, ~1,500 after single phage use, eradicated after second application	[83]
*In vitro* extracted teeth & *in vivo* rat periapical model	PEf771 and RPEf771	*E. faecalis* YN771/RYN771 in refractory periapical periodontitis	*In vitro* on extracted teeth: greater antibacterial effect within 72 h than standard root canal disinfectants; *in vivo*, phage therapy prevented death from systemic infection and eliminated periapical lesions on CT and histology	[84]
*In vitro* tooth root slices	SHEF2	*E. faecalis* biofilms on polystyrene and cross-sectional tooth roots	Eradicated biofilms on polystyrene and reduced viable biofilm on tooth slices (significant decrease by resazurin assay and microscopy)	[85]
*In vitro* & *ex vivo* root canal model	vB_ZEFP	Multidrug-resistant *E. faecalis* from infected root canals	Controlled growth and biofilm *in vitro*; prevented *ex vivo* root canal infection after treatment	[86]
*In vitro* A	S1249 (lysogenic, induced to lytic in serum)	*Aggregatibacter actinomycetemcomitans* (aggressive periodontitis)	Human serum induced lytic cycle; efficiently eliminated serum-resistant *A. actinomycetemcomitans* *in vitro*	[87]
*In vitro* implant-associated infections	Ristretto, Corretto	*Cutibacterium acnes* biofilms (implant/prosthetic infections)	Corretto achieved near-complete eradication of mature *C. acnes* biofilms across multiple strains; activity dose- and strain-dependent	[88]
*In vitro* periodontitis models (systematic review)	S1249, ΦSG005, JD-Fnp1–5, FNU1, Aabφ01, Aabφ01-1, Fnp02 (11 phages)	*Aggregatibacter actinomycetemcomitans*, *Fusobacterium nucleatum*, *Streptococcus gordonii*, *Porphyromonas gingivalis*	All included in vitro studies showed effective control of periodontal pathogens’ growth (OD and/or CFU reductions); no animal models yet	[89]
*In vitro* *S. mutans* biofilm & enamel demineralization	Phage lysin LysP53	*S. mutans* planktonic and biofilms; enamel specimens	4 μM LysP53 killed >84% planktonic *S. mutans* within 1 min at pH 4.0–6.5; daily LysP53 on biofilms reduced exopolysaccharides, virulence gene expression, live cells; prevented enamel demineralization similar to 0.12% chlorhexidine	[90]
*In vivo* mouse model (colonization)	LysP53 lysin	*S. mutans* on tooth surfaces in mice	Topical LysP53 effectively reduced *S. mutans* colonization of tooth surfaces without observable toxic effects	[90]
*In vitro* *S. mutans* isolates	Endolysins from *S. mutans* phages (crude endolysin preparations)	Clinical *S. mutans* isolates from dental caries	Endolysins showed MIC 13.6–17.8 μg/mL and MBC 17.0–22.3 μg/mL; effective *in vitro* against multidrug-resistant *S. mutans*	[91]
*In vitro* *S. mutans* biofilm & exopolysaccharide (EPS) structure	ClyR chimeric lysin (phage-derived)	*S. mutans* UA159 and gtf mutant with reduced insoluble EPS	ClyR more effective against wild-type EPS-rich biofilms; reduced biofilm and bacterial counts; demonstrated key role of water-insoluble EPS in mediating lysin binding and antibacterial activity	[92]
*In vivo* rat caries model	ClyR lysin	*S. mutans* / *S. sobrinus* single or mixed infections	Continuous ClyR use markedly decreased caries rate in rat models, with selective action on cariogenic streptococci and sparing commensals	[93]

**Table 3:** Current evidence and future perspectives on the role of the oral virome in dental caries.

Topic	Well Supported (Current Evidence)	Emerging Evidence	Speculative / Research Gaps
Oral virome composition	The oral cavity harbors diverse bacteriophages and eukaryotic viruses with niche-specific distribution.	Individual virome signatures may reflect oral health status.	Longitudinal stability and clinical significance require validation.
Role of herpesviruses	Herpesviruses modulate host immunity and contribute to oral inflammation.	They may indirectly favor cariogenic bacterial activity by altering the oral microenvironment.	Their direct contribution to dental caries remains unproven.
Bacteriophages as ecological regulators	Phages regulate bacterial populations through lytic predation and lysogeny, influencing microbial ecology.	Phage-driven modulation of plaque dysbiosis may contribute to caries development.	The ecological mechanisms governing phage–bacteria interactions *in vivo* remain incompletely understood.
Virome-based biomarkers	Metagenomic studies demonstrate distinct phage profiles associated with caries.	Salivary phage signatures may enable early diagnosis and risk assessment.	Prospective clinical validation and standardized diagnostic platforms are lacking.
Phage therapy	Lytic phages effectively reduce *Streptococcus mutans* growth and biofilms *in vitro* and in animal models.	Topical phage formulations (*e.g.*, varnishes and sustained-release systems) show promising preclinical efficacy.	Human clinical trials demonstrating safety and efficacy are currently unavailable.
Phage-derived enzymes (enzybiotics)	Endolysins and depolymerases exhibit potent antibiofilm activity against cariogenic bacteria.	Engineered lysins may provide highly targeted antimicrobial therapy.	Clinical translation and optimized delivery systems remain under development.
Phage-derived peptides	Experimental studies demonstrate selective activity against cariogenic bacteria and biofilms.	Smart peptide delivery systems may improve specificity and efficacy.	Clinical efficacy, long-term safety, and regulatory approval remain to be established.
Future therapeutic strategy	Ecological modulation of the oral microbiome is increasingly recognized as a promising approach.	Integration of phages, enzybiotics, and precision diagnostics may enable personalized caries management.	Large-scale clinical trials and regulatory frameworks are needed before routine clinical implementation.
